# Deer Horn Sign in Congestive Hepatopathy Due to Heart Failure

**DOI:** 10.3390/reports8020079

**Published:** 2025-05-23

**Authors:** Thomas Ferenc, Andro Matković, Jelena Svetec, Filip Brkić, Tomica Bratić, Vitorio Perić, Vinko Vidjak

**Affiliations:** 1 Department of Diagnostic and Interventional Radiology, Merkur University Hospital, 10000 Zagreb, Croatiatomica.bratic@gmail.com (T.B.); vitorioperic@gmail.com (V.P.);; 2 School of Medicine, University of Zagreb, 10000 Zagreb, Croatia

**Keywords:** congestive hepatopathy, congestive heart failure, deer horn sign, ultrasonography

## Abstract

**Background and Clinical Significance:** The deer horn sign is an ultrasonographic (US) finding suggesting congestive hepatopathy. It is composed of dilated intrahepatic inferior vena cava (IVC) representing the deer’s head and dilated hepatic veins (HVs) representing its horns. **Case Presentation:** A 72-year-old female patient presented with a one-week history of dull pain in the right upper abdominal quadrant. Her medical records showed that she had previously experienced cardiovascular problems; however, she is without any recent heart failure symptoms. The transabdominal US demonstrated the deer horn sign and hemodynamic changes in the hepatic venous drainage, which is suggestive of congestive hepatopathy. An echocardiogram revealed congestive heart failure with a preserved ejection fraction, mild-to-moderate mitral and tricuspid valve insufficiency, and severe aortic valve stenosis with mild aortic valve insufficiency. **Conclusions:** The definite diagnosis of heart failure is based on clinical and laboratory features; however, this sign may be helpful for diagnosis in emergency settings.

## 1. Introduction and Clinical Significance

**Figure 1:**
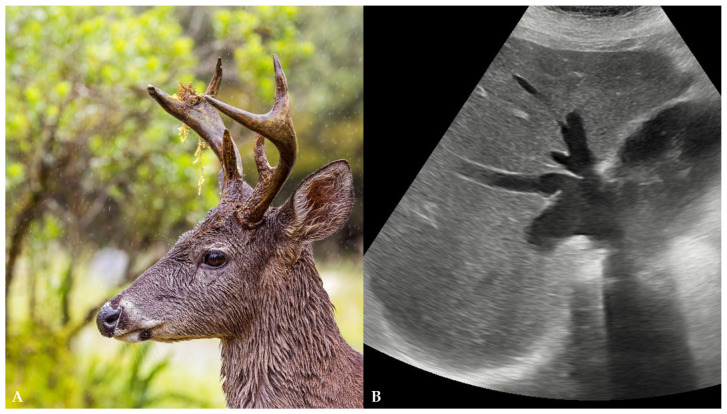
(**A**) An image of a white-tailed deer in Chingaza National Park, Colombia [author Charles J. Sharp; available at https://commons.wikimedia.org/wiki/File:White-tailed_deer_(Odocoileus_virginianus_goudotii)_male_head_Chingaza.jpg (accessed on 24 March 2025)]. (**B**) An ultrasound image of the deer horn sign where the dilated intrahepatic inferior vena cava represents the deer’s head and dilated hepatic veins represent the deer’s horns.

Heart failure (HF) is a complex clinical syndrome that results from any structural or functional impairment of ventricular filling or ejection of blood with a wide range of possible etiological factors (e.g., ischemic, hypertensive, valvular, systemic, or congenital heart diseases). Subsequent alterations in hemodynamics can affect the liver and lead to congestive hepatopathy, indicated by the dilatation of hepatic veins (HVs) and intrahepatic inferior vena cava (IVC) due to venous stasis and elevated central venous pressure [1]. Chronic congestive hepatopathy may lead to hepatocyte injury and the development of cardiac fibrosis and, ultimately, cirrhosis [1,2]. In an emergency setting, transabdominal ultrasound (US) is often the first imaging modality for the evaluation of patients who present with HF and abdominal pain and have abnormal liver function tests [1,2,3]. Herein, we report a case of a female patient who presented with a US finding of deer horn sign (Figure 1), which was suggestive of congestive hepatopathy.

## 2. Case Presentation

A 72-year-old female patient presented to the emergency department (ED) with a one-week history of dull pain in the right upper abdominal quadrant, followed by a loss of appetite and nausea, without any significant weight loss in the past 3 months. At the time of the visit, the patient’s BMI was 31.4. The patient’s medical records showed that she experienced two NSTEMI episodes with percutaneous coronary artery stenting (in 2016 and 2022) and was also previously diagnosed with moderate aortic valve stenosis, mild-to-moderate mitral valve insufficiency, subclavian steal syndrome, and small intracerebral aneurysms of the basilar artery and right middle cerebral artery. She underwent cholecystectomy in 2020. At the time of the ED visit, she was under treatment for arterial hypertension and atrial fibrillation without any recent heart failure symptoms. During the clinical examination, the patient complained of dull pain in the right upper abdominal quadrant, particularly during deep palpation of the abdomen. Elevated values in laboratory blood analysis are displayed in Table 1. Laboratory tests were otherwise unremarkable.

**Figure 2:**
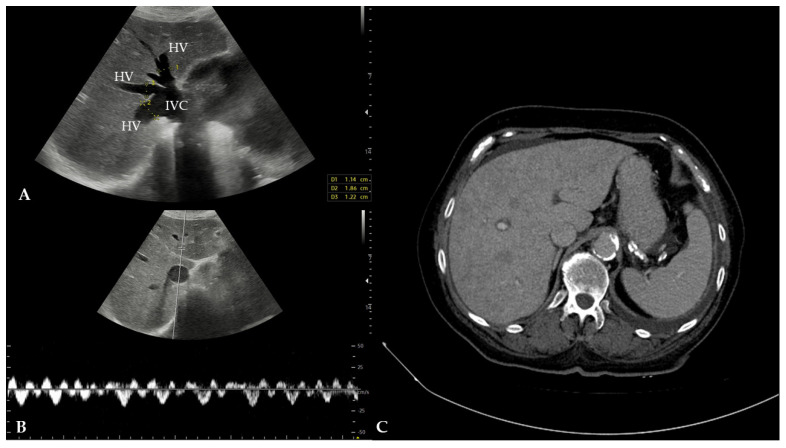
(**A**) The dilated hepatic vein (HV) lumen ranged from 12 to 18 mm in diameter. The intrahepatic inferior vena cava (IVC) was also dilated (31 mm in diameter; measurement not annotated in the image). (**B**) Spectral waveform analysis of the patient’s hepatic veins revealed the loss of a regular triphasic flow pattern, with an impression of a prominent a wave and v wave. These findings represent hemodynamic changes in the hepatic venous system that were highly suggestive of congestive hepatopathy. (**C**) Contrast-enhanced CT of the upper abdomen revealed lobulated liver contours and a “nutmeg” appearance of the parenchyma, which is indicative of chronic hepatic vein congestion.

**Figure 3:**
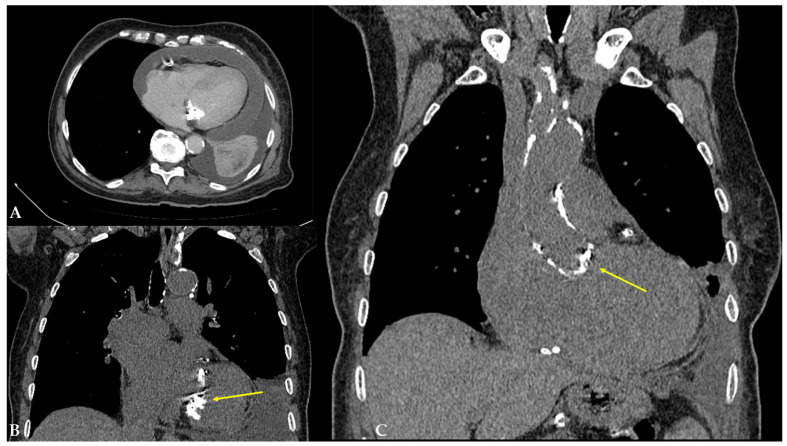
(**A**) Contrast-enhanced CT of the lung basis displayed moderate pericardial effusion and pleural effusion. (**B**) Non-contrast-enhanced CT of the thorax revealed an enlarged heart with extensive calcifications of the tricuspid and mitral valves (arrow). (**C**) Non-contrast-enhanced CT of the thorax demonstrated a highly calcified thoracic aorta wall and aortic valve (arrow).

The patient was then referred for a transabdominal US under the suspicion of biliary pathology or fluid collection, which was excluded during the examination. The US demonstrated mildly irregular liver contours, dilated HVs, and intrahepatic IVC with a characteristic deer horn sign and hemodynamic changes in the hepatic venous system, which is highly suggestive of congestive hepatopathy (Figure 2). The US also revealed moderate ascites, moderate pleural effusion on the left side, and severe pericardial effusion. An echocardiogram revealed a dilated left atrium (4.6 cm; normal range: 1.9–4.0 cm), a dilated right ventricle (3.4 cm; normal range: 0.7–2.6 cm in diastole), and concentric hypertrophy of the left ventricle with a preserved ejection fraction (70%) and no regional contractility disorders. It measured 4.5 cm in diameter (normal range: 3.5–5.6 cm) with thickening of the interventricular septum (1.6 cm; normal range: 0.7–1.2 cm) and the posterior wall of the left ventricle (1.6 cm; normal range: 0.7–1.2 cm). The echocardiogram also revealed mild-to-moderate mitral and tricuspid valve insufficiency, as well as severe aortic valve stenosis with mild aortic valve insufficiency. The severity of the pericardial effusion was downgraded to moderate compared to the transabdominal US. Echocardiographic findings correlated with congestive heart failure with a preserved ejection fraction. The internal medicine specialist then referred the patient to a contrast-enhanced abdominal computed tomography (CT) due to elevated pancreatic enzymes; however, no signs of pancreatic inflammation were found. The patient was hospitalized later that day for an extensive cardiac workup due to preparation for aortic valve replacement. In the upcoming days, coronary angiography and non-contrast-enhanced chest CT were performed and showed a highly calcified left coronary artery and thoracic aorta, respectively (Figure 3). The patient was discharged from the hospital in good general condition and was referred to a cardiac surgeon for further evaluation and treatment arrangements. The patient’s primary diagnosis was congestive heart failure due to severe aortic stenosis. At the same time, the imbalance in the autonomic nerve system, sodium retention, and fluid accumulation resulted in subsequent peripheral and splanchnic congestion.

## 3. Discussion

One of the transabdominal US signs that may raise suspicion for congestive hepatopathy is the deer horn sign, with the dilated intrahepatic IVC representing the deer’s head and dilated HVs representing the horns [3,4,5]. The normal right HV diameter is 5.6–6.2 mm and is increased to 8.8 mm in the case of chronic HF and to 13.3 mm in the presence of chronic HF with pleural effusion [6]. The abnormal IVC diameter is over 21 mm [7]. Depending on the plane of image acquisition and interpretation by the individual performing the US examination, other signs have also been introduced to describe the exact appearance, such as the “Playboy Bunny” sign and the “Moose Head” sign [1,2,5]. Bartrum and Crow first mentioned the Playboy Bunny sign in 1983 to describe hepatic vein confluence in healthy individuals [1]. Some authors believe that the appearance of the deer horn sign indicates more severe congestion due to more dilated hepatic veins [3]. Other transabdominal sonographic findings in patients with HF may be irregular liver contours with altered parenchymal echogenicity, gallbladder wall thickening due to edema, and pleural and pericardial effusion [2,3,5]. Other possible diagnoses that should be excluded are Budd–Chiari syndrome (BCS) and sinusoidal obstruction syndrome (SOS). In patients with BCS, depending on the duration of the disease, some of the typical US findings are non-visualization and thrombosis of HVs with intrahepatic collateral pathways, enlargement of the caudate lobe, ascites, and splenomegaly [8]. Some typical US findings in patients with SOS are hepatomegaly, splenomegaly, gallbladder thickening, increased portal vein diameter, decreased HV diameter, and ascites [9].

The presence of severe liver steatosis may influence the interpretation of this sign due to the significant attenuation of US waves and the unclear visualization of intrahepatic vessel borders. The sensitivity and specificity of the deer horn sign for diagnosing congestive hepatopathy, as well as the precise learning curve for sonographers to detect it, have yet to be determined. There is a list of various signs in abdominal radiology. It may be challenging to memorize them, especially if similar signs have different names, leading to terminology confusion. However, for inexperienced users (e.g., residents) or visual learners, it can be beneficial to become familiar with the most common signs and their characteristic images, which can help increase the accuracy of examinations and narrow down the differential diagnosis.

## 4. Conclusions

The visualization of the deer horn sign is a US finding suggestive of congestive hepatopathy due to congestive heart failure. Although the definitive diagnosis of HF is based on clinical and laboratory features, this sign may raise suspicion of HF and facilitate the correct diagnosis in an emergency setting.

## Figures and Tables

**Table 1:** Laboratory blood analysis and reference values.

Blood Test	Value	Reference Value
Glucose	8.3	4.4–6.4 mmol/L
AST	89	11–34 U/L
ALT	73	8–41 U/L
GGT	42	9–35 U/L
Total bilirubin	41	3–20 μmol/L
Creatinine	110	49–90 μmol/L
Alfa-amylase	112	23–91 U/L
Lipase	118	13–60 U/L
CRP	99	<5 mg/L
NT-proBNP	14235.8	<125 ng/L
High-sensitivity troponin I	22.1	15.6 ng/L for those older than 20 years of age

## Data Availability

Data are contained within the article, and further inquiries can be directed to the corresponding author.
